# Pilot study on cultural and metagenomic analysis of bile and biliary stentslead to unveiling the key players in stent occlusion

**DOI:** 10.1038/s41598-024-51480-2

**Published:** 2024-02-09

**Authors:** Margherita Cacaci, Flavio De Maio, Maria Valeria Matteo, Brunella Posteraro, Maura Di Vito, Giulia Menchinelli, Andrea Tringali, Francesca Romana Monzo, Riccardo Torelli, Guido Costamagna, Cristiano Spada, Francesca Bugli, Maurizio Sanguinetti, Ivo Boskoski

**Affiliations:** 1 Department of Basic Biotechnological Sciences, Intensive and Perioperative Clinics, Università Cattolica del Sacro Cuore, 00168 Rome, Italy; 2 Department of Laboratory and Infectious Sciences, Fondazione Policlinico Universitario A. Gemelli IRCCS, Largo A. Gemelli 8, 00168 Rome, Italy; 3 Digestive Endoscopy Unit, Fondazione Policlinico Universitario Agostino Gemelli IRCCS, Rome, Italy; 4 Center for Endoscopic Research Therapeutics and training (CERTT), Università Cattolica del Sacro Cuore, Rome, Italy; 5 Department of Abdominal and Endocrine Metabolic Medical and Surgical Sciences, Fondazione Policlinico Universitario A. Gemelli IRCCS, 00168 Rome, Italy

**Keywords:** Microbiology, Gastroenterology

## Abstract

Endoscopic Retrograde Cholangio-Pancreatography (ERCP) with biliary stenting is a minimally invasive medical procedure employed to address both malignant and benign obstructions within the biliary tract. Benign biliary strictures (BBSs), typically arising from surgical interventions such as liver transplants and cholecystectomy, as well as chronic inflammatory conditions, present a common clinical challenge. The current gold standard for treating BBSs involves the periodic insertion of plastic stents at intervals of 3–4 months, spanning a course of approximately one year. Unfortunately, stent occlusion emerges as a prevalent issue within this treatment paradigm, leading to the recurrence of symptoms and necessitating repeated ERCPs. In response to this clinical concern, we initiated a pilot study, delving into the microbial composition present in bile and on the inner surfaces of plastic stents. This investigation encompassed 22 patients afflicted by BBSs who had previously undergone ERCP with plastic stent placement. Our preliminary findings offered promising insights into the microbial culprits behind stent occlusion, with Enterobacter and Lactobacillus spp. standing out as prominent bacterial species known for their biofilm-forming tendencies on stent surfaces. These revelations hold promise for potential interventions, including targeted antimicrobial therapies aimed at curtailing bacterial growth on stents and the development of advanced stent materials boasting anti-biofilm properties.

## Introduction

Endoscopic Retrograde Cholangio-Pancreatography (ERCP) with biliary stenting is a minimally invasive procedure widely performed to treat both malignant and benign obstructive conditions of the biliary tract^1^. Benign biliary strictures (BBSs) are mainly a consequence of postsurgical injuries, such as liver transplant and cholecystectomy, along with chronic inflammatory disorders such as chronic pancreatitis^2^. Currently, the gold standard treatment for BBSs is the insertion of as many plastic stents as possible every 3–4 months over a period of about 1 years^1,3^, or in some conditions, fully covered self-expandable metals stents (FCSEMS)^1^. The stent change interval is based on the predicted median patency of 80–126 days for a single plastic stent^4,5^, or on average 6 months for FCSEMS^1^. Since the risk of occlusion is related to the diameter of the stent, FCSEMS were introduced to expand the stent diameter, thus prolonging the patency and reducing the frequency of stent substitutions^1^. However, these stents can be used only in distal BBSs and have a high migration rate^6^. Moreover, despite the longer dwelling time, FCSEMS are far more costly than plastic stents and sometimes the removal can be troublesome^7,8^. As it concerns malignant biliary strictures, the use of plastic, covered or uncovered SEMS depends on life expectancy, tumour status (operable or non-operable), the need for adjuvant or neoadjuvant treatments etc^9^.

The primary cause for plastic or FCSEMS stents obstruction in BBSs is the development of sludge, which is composed of a mixture of microorganisms, fibres, proteins, calcium bilirubinate, and calcium palmitate^10,11^, while in malignant biliary strictures, the cause of stent occlusion is also in growth through the meshes of the stent. Although the development of biliary sludge is probably a multifactorial event, bacterial colonisation and biofilm formation are recognised as leading factors in biliary plastic and FCSEMS blockage^12^. Several papers have examined the phenomenon of stent occlusion, evaluating causative factors involved in the clogging process and microbial biofilm formation^10–14^. The longer dwelling time of FCSEMS is only due to their inner diameter which is up to 10 mm versus approximately 3 mm of plastic stents. Moreover, the mean patency of FCSEMS is 6 months versus up to 3 months of plastic stents. Plastic stents are made of material that cannot prevent the creation of bacterial biofilm and sludge. The main solution to the problem would be the development of innovative plastic stents that possess inherent antimicrobial properties and demonstrate the potential to mitigate biofilm formation, thus offering a promising solution to this long-standing problem. The implementation of such advancements in stent design could significantly improve patient outcomes and reduce the burden of associated complications. In this view, little is known about the microbiota associated with benign biliary strictures and, particularly, the possible microbial communities responsible for biofilm formation and stent occlusion. As far as we know, there are no published studies analysing the microbiota inside biliary stents using the Next Generation Sequencing (NGS) based genotyping technique. To gain insight into the microorganisms involved in biliary stent occlusion, a pilot study was conducted to characterise the microbial composition on both bile and the inner surface of plastic stents using traditional cultural techniques and NGS-based genotyping. The results of this study, although preliminary, allowed us to identify specific bacterial species and biofilm-forming communities that contribute to stent occlusion. This knowledge can help in developing strategies for preventing or managing stent occlusion, such as targeted antimicrobial therapy or the development of improved stent materials with reduced biofilm formation.

## Materials and methods

### Study population

This prospective pilot study was performed at Fondazione Policlinico Universitario A. Gemelli IRCCS in Rome from July 2019 to February 2021. Over this period, selected patients with BBSs requiring emergency or elective bile stent exchange/withdrawal were consecutively recruited. Patients with neoplastic biliary stritrures and BBSs following orthotopic liver transplantation were excluded. Due to the nature of this pilot study, we decided to enroll 22 patients (respecting the inferior number of 12 patients suggested by Julious in his study “*Sample size of 12 rule of thumb for a pilot study”*^15^. Clinical data of each patient were recorded in terms of age, sex, aetiology of BBSs, indwelling time of stents, the clinical setting of stents replacement (elective/emergency), number of previous stents exchanges, the use of antibiotic prophylaxis before ERCP, and the use of antibiotics in the previous six months.

The institutional ethical committee approved this clinical investigation (protocol number 32041/19, ID 2643)). The study was performed in accordance with the ethical standards as laid down in the 1964 Declaration of Helsinki and its later amendments or comparable ethical standards.

### Bile and stents sampling and microbiological analysis

Prior to the removal of the plastic stent, its correct positioning was confirmed using fluoroscopy to ensure that the stents were in the correct place. The duodenoscope was then carefully introduced and positioned in front of the papilla without the use of suction, similar to the technique used for the *Helicobacter pylori* microbiological test. Next, the stents were safely and effectively removed using an endoscopic procedure. A sterile snare was employed to carefully grasp the stents, and they were subsequently pulled into the operative channel of the endoscope.

From each stent, the corresponding bile drops were carefully collected. Stent and bile samples collected were placed in sterile tubes and transported to the Microbiology lab within two hours from the extraction. Bile samples were cultured in aerobic conditions using the following aerobic plates: Chocolate agar (PVX), Tryptone Soy Agar (TSA), MacConkey agar, Columbia sheep blood agar (CNA), (OXOID, Thermo Fisher, UK), and candida bromocresol green (BCG agar), (BD, UK) and in anaerobic conditions on Shaedlaer agar, (SCH) and CNA agar and incubated in aerobic and anaerobic atmosphere at 37 °C for 24h and 48 h, respectively. Proximal and distal stent extremities were cut off by 1 cm to prevent potential contaminants. Then, stents were placed in a sterile falcon tube with 50 ml of phosphate-buffered saline (PBS) solution and sonicated in ultrasound bath (BactoSonic, Bandelin) at 40 kHz for 45 min. Samples were then vortexed for 30 s and stents removed. Falcon tubes were centrifugated at 4.500 rpm for 20 min and pellets resuspended in 20 ml of Thioglycolate broth (Merk), divided in two 10 ml aliquots that were incubated either in aerobic conditions or anaerobic conditions, as previously described. Tube grown in aerobic condition were then plated on PVX, TSA, MacConkey, CNA and BCG while the tubes grown in anaerobic condition were plated in SCH and CNA agar, as previously described.

### Strains identification and antimicrobial susceptibility testing

Bacteria and fungi grown from bile and stents samples were identified by matrix-associated laser desorption/ionization-time of flight mass spectrometer (MALDI-TOF, Bruker Corporation, Billerica, U.S.A.).

Antimicrobial Susceptibility Testing (AST) were performed by the VITEK® 2 system according to the manufacturer’s instructions, using the software version 7.01 and the AST-N379 cards for Gram-negative bacteria, the AST-P658 for *Enterococcus* spp. and the AST-ST for *Streptococcus* spp. Minimum inhibitory concentrations (MICs) were established by the instrument and categorized in S (susceptible), R (resistant) and I (Susceptible, increased exposure) following EUCAST breakpoints Version 13.1, 2023. (http://www.eucast.org).

### DNA extraction, library preparation and sequencing

Bile and stent specimens were kept on ice from the time of collection until transport to Microbiology laboratory and stored at − 80 °C until processing. Total DNA extraction was processed in a strictly controlled, separate and sterile workplace. Briefly, 500 µl of each bile sample were centrifuged, and the recovered pellet resuspended with a sterile phosphate buffer. This suspension and the culture obtained from each stent, cultivated either in aerobic or anaerobic conditions, were used to extract DNA by using DNeasy PowerSoil Kit (Quiagen, Germany) according to manufacturer’s instruction. Quality and concentration of the extracted DNA was evaluated for each sample by agarose gel electrophoresis (Life Technologies) and Qubit 4.0 fluorometer, with ds DNA High sensitivity assay (Life Technologies), respectively.

V5-V6 hypervariable regions of the 16S rRNA gene were amplified^16,17^ . Amplicons were purified by using Agencourt AMPure XP beads (Beckman Coulter) and then barcoded with Nextera XT index (Illumina). Each indexed amplicon was equimolarly diluted, and the final pool was properly prepared for the paired ends sequencing^18^ (2 × 250 bp, v2 chemistry, Illumina) on the Illumina MiSeq instrument (Illumina, San Diego, CA). To increase degree of base diversity, the internal control PhiX v3 (Illumina) was added to the library.

### Sequencing and data analysis

16S-rRNA-gene amplicon sequencing reads were processed with QIIME2 plugins v2019.7^18^. We carried out a demultiplexing and a quality inspection of paired end reads. Illumina adapter sequence (5’ - CTGTCTCTTATACACATCT - 3’) was trimmed before denoising paired-ends reads with QIIME2’s DADA plugin, following default settings and adjusting the number of bases trimmed at the 5’- and 3’- ends to remove non biological primer sequences and to merge reads^19^. Denoising output resulted in a ≈ 70% of good, merged reads and Amplicon sequence variants (ASVs) were summarized. We performed a taxonomic classification using the VSEARCH global consensus alignment and reference sequences previously retrained from SILVA132 database^20^ (sequences were clustered at 99% sequence similarity). A phylogenetic tree of the resulting ASVs was obtained. Finally, a biological observation matrix (BIOM) was generated merging ASVs table and taxonomic information.

Further analysis was carried out using R v4.0.2 (https://www.rstudio.com/) and phyloseq package for downstream analyses in-house analysis pipeline^21^.

Briefly, unassigned ASVs were removed, and a final dataset was generated applying a taxonomic filtering which removed low prevalent taxa (Chloroflexi, Cyanobacteria, Deinococcus-Thermus, Lentisphaerae, Spirochaetes, Patescibacteria, Schekmanbacteria and Tenericutes). Pre-processing process was completed removing taxa following a prevalence threshold as 1% of the total samples. To quantify diversity and equitability within each sample (alpha diversity), we used the ultimate dataset to compute Inverse Simpson diversity index and Pielou’s evenness, respectively. Beta diversity metric was assessed by using weighted UniFrac distance^22^.

Differences according to alpha diversity metrics were assessed using the Kruskal–Wallis test, whereas those according to beta diversity metric was assessed using the permutational multivariate analysis of variance (PERMANOVA). To minimize the effect of differences in sequencing depth, we computed rarefaction curves and scaled at 50,000 reads of the sequencing depth before measuring alpha and beta diversities. Relative abundances were computed at phylum- and genus- level and statistical significance assessed by Kruskal–Wallis test. Finally, microbial biomarkers were determined by DESeq2 analysis. In all analyses, statistical significance was set at a *p* < 0.05^23^.

### Ethical statement

All patients involved in the study gave their informed consent. The Ethics Committee of the Fondazione Policlinico A. Gemelli, IRCCS-Catholic University of Sacred Heart of Rome approved the study (Prot. 32041/19; ID:2643). All methods were performed in accordance with the relevant guidelines and regulations.

## Results

### Study population

Twenty-two patients (41% female, 59% male, median age 65 years, range: 42–82) affected by benign biliary strictures (BBSs) requiring endoscopic stent replacement, were enrolled from July 2019 to February 2021. Baseline characteristics of the patients are summarized in Table 1 and extensively described in Table S1.

**Table 1:** Baseline characteristics of patients.

Number of patients, *n*	22
Male, *n* (%)	13 (59.1%)
Median age, *n* (Q1-Q3)	65 (54–70)
Indication to stent therapy:	
Biliary stricture after cholecystectomy, *n*	18
Biliary stricture after severe acute pancreatitis, *n*	1
Idiopathic biliary stricture, *n*	3
Median indwelling time in days (Q1-Q3)	120 (99–161)
Elective stents extraction, *n*	19
Emergency stents extraction, *n*	3
Median number of ERCP with stent placement (Q1-Q3)	3 (2–4)
Antibiotic prophylaxis, *n*	Amoxicillin/Clavulanic Acid: 15 Ciprofloxacin + Metronidazole: 1 Piperacillin/Tazobactam: 1 Ceftriaxone: 1 None: 4
Antibiotic therapy in the previous 6 months, *n*	Amoxicillin/Clavulanic Acid: 14 Ciprofloxacin + Metronidazole: 1 Piperacillin/Tazobactam: 2 Data not available: 3 None: 2

The indication to endoscopic biliary stenting was biliary stricture after cholecystectomy in 18 patients (81.8%), biliary stenosis after necrotizing acute pancreatitis in one patient (4.6%) and idiopathic stricture in the remaining 3 patients (13.6%). Emergency stents exchange was necessary in 3 patients because of cholangitis and in one patient due to obstructive jaundice. The median duration of stents in place was 120 days. Empirical antibiotic prophylaxis was administered before ERCP in most patients, with amoxicillin/clavulanic acid being the most frequently used and with at least one dose (Tables 1 and 2). The two patients with acute cholangitis received a course of antibiotic treatment with ciprofloxacin combined with metronidazole and ceftriaxone, respectively. The patient with obstructive jaundice received prophylaxis with amoxicillin/clavulanic acid before emergency ERCP. Most patients received antibiotics in the previous six months, mainly at the time of the previous ERCP (Tables 1 and S1).

**Table 2:** Microorganisms identified by MALDI-TOF after culture of bile (*n* = 22) and stent (*n* = 22) samples.

Microorganisms	Site of collection		*P value*
	Bile, *n* (%)	Stent, *n* (%)	
Gram-positive aerobic bacteria			
*Enterococcus* spp.	48 (24.5)	49 (28.0)	0.44
*Enterococcus.* *faecalis*	18 (9.2)	17 (9.7)	0.59
*Enterococcus* *faecium*	13 (6.6)	12 (6.9)	0.70
*Enterococcus* *avium*	4 (2.0)	6 (3.4)	0.31
*Enterococcus* *gallinarum*	4 (2.0)	5 (2.9)	0.49
*Enterococcus* *casseliflavus*	4 (2.0)	6 (3.4)	0.36
*Enterococcus* *hirae*	1 (0.5)	1 (0.6)	0.89
*Enterococcus.* *raffinosus*	3 (1.5)	1 (0.6)	0.41
*Enterococcus*. *maleodorant*	1 (0.5)	1 (0.6)	0.89
*Streptococcus* spp.	8 (4.1)	2 (1.1)	0.17
*Streptococcus* *anginosus*	4 (2.0)	1 (0.6)	0.28
*Streptococcus* *massiliensis*	1 (0.5)	1 (0.6)	0.89
*Streptococcus* *constellatus*	1 (0.5)	0	–
*Streptococcus* *gallolyticus*	2 (1.0)	0	–
Others	15 (7.7)	25 (14.3)	0.37
*Lactobacillus* spp	14 (7.1)^a^	24 (13.7)^b^	0.24
*Cellulomonas* *denverensis*	1 (0.5)	1 (0.6)	0.89
Gram-negative aerobic bacteria			
Enterobacterales	65 (33.2)	61(34.9)	0.22
*Citrobacter* *freundii*	4 (2.0)	5 (2.9)	0.49
*Citrobacter* *koseri/brakii*	3 (1.5)	5 (2.9)	0.32
*Enterobacter* *bugandensis*	0	1 (0.6)	–
*Enterobacter* *cloacae*	5 (2.6)	3 (1.7)	0.69
*Escherichia* *coli*	16 (8.2)	16 (9.1)	0.51
*Hafnia* *alvei*	2 (1.0)	1 (0.6)	0.67
*Klebsiella* *oxytoca*	9 (4.6)	6 (3.4)	0.69
*Klebsiella* *pneumoniae*	15 (7.7)	16 (9.1)	0.40
*Klebsiella* *varicola*	3 (1.5)	1 (0.6)	0.41
*Morganella* *morganii*	4 (2.0)	4 (2.3)	0.74
*Proteus* *hauserii*	1 (0.5)	0	–
*Proteus* *mirabilis*	2 (1.0)	2 (1.1)	0.85
*Proteus* *vulgaris*	1 (0.5)	1 (0.6)	0.89
Others	5 (2.6)	1 (0.6)	0.17
*Aeromonas* *veronii*	1 (0.5)	0	–
*Aeromonas* *hydrophila*	1 (0.5)	0	–
*Pseudomonas* *aeruginosa*	2 (1.0)	1 (0.6)	0.67
*Serratia* *marcescens*	1 (0.5)	0	–
Gram-positive anaerobic bacteria			
Bifidobacterium spp.	5 (2.6)	1 (0.6)	0.17
*Bifidobacterium* *animalis*	3 (1.5)	0	–
*Bifidobacterium* *breve*	1 (0.5)	0	–
*Bifidobacterium* *pseudolongum*	1 (0.5)	1 (0.6)	0.89
Clostridium spp.	10 (5.1)	9 (5.1)	0.79
*Clostridium* *clostridioforme*	1 (0.5)	2 (1.1)	0.46
*Clostridium* *difficile*	1 (0.5)	1 (0.6)	0.89
*Clostridium* *perfrigens*	6 (3.1)	5 (2.9)	0.99
*Clostridium* *ramosum*	1 (0.5)	1 (0.6)	0.89
*Clostridium* *sordellii*	1 (0.5)	0	–
Others	5 (2.6)	5 (2.9)	0.91
*Actynomices* *dentalis*	0	1 (0.6)	–
*Anaerococcus* *hydrogenalis*	1 (0.5)	1 (0.6)	0.89
*Facklamia* *hominis*	1 (0.5)	0	–
*Pediococcus* *acidilactici*	1 (0.5)	2 (1.1)	0.46
*Peptoniphilus* *harei*	1 (0.5)	0	–
*Propionibacterium* *aidipropionae*	0	1 (0.6)	–
*Slackia* *exigua*	1 (0.5)	0	–
Gram-negative anaerobic bacteria			
Bacteroides spp.	4 (2.0)	2 (1.1)	0.59
*Bacteroides* *fragilis*	1 (0.5)	0	–
*Bacteroides* *ovatus*	1 (0.5)	0	–
*Bacteroides* *tethaiotaomicron*	1 (0.5)	1 (0.6)	0.89
*Bacteroides* *vulgatus*	1 (0.5)	1 (0.6)	0.89
Veillonella spp.	3 (1.5)	4 (2.3)	0.53
*Veillonella* *atypica*	0	1 (0.6)	–
*Veillonella* *parvula*	3 (1.5)	3 (1.7)	0.82
Others	3 (1.5)	1 (0.6)	0.41
*Acidaminococcus* *intestini*	1 (0.5)	1 (0.6)	0.89
*Parabacteroides* *buccae*	1 (0.5)	0	–
*Parabacteroides* *distasonis*	1 (0.5)	0	–
Yeasts			
Candida spp.	23 (11.7)	14 (8.0)	0.39
*Candida* *albicans*	17 (8.7)	13 (7.4)	0.89
*Candida* *glabrata*	4 (2.0)	1 (0.6)	0.28
*Candida* *krusei*	1 (0.5)	0	–
*Candida* *tropicalis*	1 (0.5)	0	–
Others	2 (1.0)	1 (0.6)	0.67
*Pichia* *manshurica*	1 (0.5)	0	–
*Saccaromyces* *cerevisiae*	1 (0.5)	1 (0.6)	0.89

^*a*^*Lactobacillus* *agilis* (n = 1), *L.* *curvatus* (n = 1), *L.* *gasseri* (n = 3*), L.* *johnsonii* (n = 2), *L.* *mucosae* (n = 4), *L.* *oris* (n = 1), *L.* *reuteri* (n = 2).

^*b*^*Lactobacillus* *agilis* (n = 2), *L.* *curvatus* (n = 1), *L.* *gasseri* (n = 5), L*.* *johnsonii* *(n* = *6*), *L.* *mucosae* (n = 4), *L.* *oris* (n = 2), *L.* *pseudolongum* (n = 1), L. reutori (n = 1), *L.* *vaginalis* (n = 2).

### Microbiological characterization of bile and stent samples

**Figure 1:**
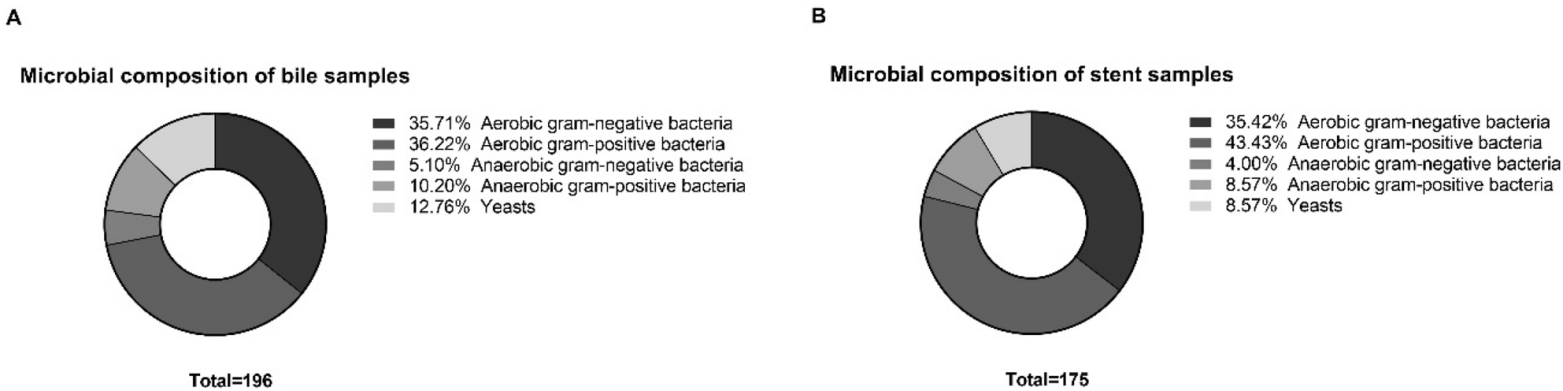
Microbial composition (percentage) of bile and stent samples identified by cultural techniques and MALDI-TOF MS.

Bile and relative stents, grown in aerobic and anaerobic conditions, were microbiologically analysed. All the samples showed a polymicrobial colonization. Up to 16 species were identified in a single sample. Overall, 196 and 175 microorganisms were isolated from bile and stents samples, respectively. Precisely, 36.2% and 43.4% were gram positive bacteria, 35.7% and 35.4% were gram-negative bacteria, 10.2% and 8.6% were anaerobic gram-positive bacteria, 5.1% and 4.0% by anaerobic gram-negative bacteria while yeasts, represented 12.8% and 8.6% in bile and stent samples, respectively (Fig. 1).

The predominant species isolated either in bile and stent samples were *Lactobacillus* spp (7.1 and 13.7% in bile and stent samples, respectively), *Enterococcus faecalis* (9.2% and 9.7%), followed by *Escherichia coli* (8.2 and 9.1%), *Klebsiella pneumoniae* (7.7% and 9.1%, respectively) and *Enterococcus faecium* (6.6% and 6.9%). Among anaerobic gram-positive bacteria, the most isolated species resulted to be *Clostridium* spp (5.1% and 5.1%), particularly *C. perfrigens* (3.1% and 2.9%) while among gram-negative anaerobic bacteria, the most encountered species was Bacteroides *spp.* (2.0% and 1.1%), without any differences between the single species. Finally, the most isolated yeast was *Candida* spp. (11.7% and 8.0%), particularly *Candida albicans* (8.7% and 7.4%) (Table 2).

Bile and stent samples showed a common microbial flora, but bile samples were found to be richer in terms of number of isolated microorganisms except for *E. coli*, *K. pneumoniae* and *Lactobacillus* spp. which appeared to be more prevalent in the stent samples compared to the corresponding bile samples, but no significative statistical differences were detected between the two groups.

All the aerobic bacteria, isolated from bile and stent samples, were tested for their susceptibility to the antibiotic used in clinical practice. Almost all the tested isolates showed variable susceptibility profiles with some resistances to different classes of antibiotics depending on the species. No differences have been detected in the antimicrobial susceptibility profile of the same species isolated from the bile and the corresponding stent samples.

**Figure 2:**
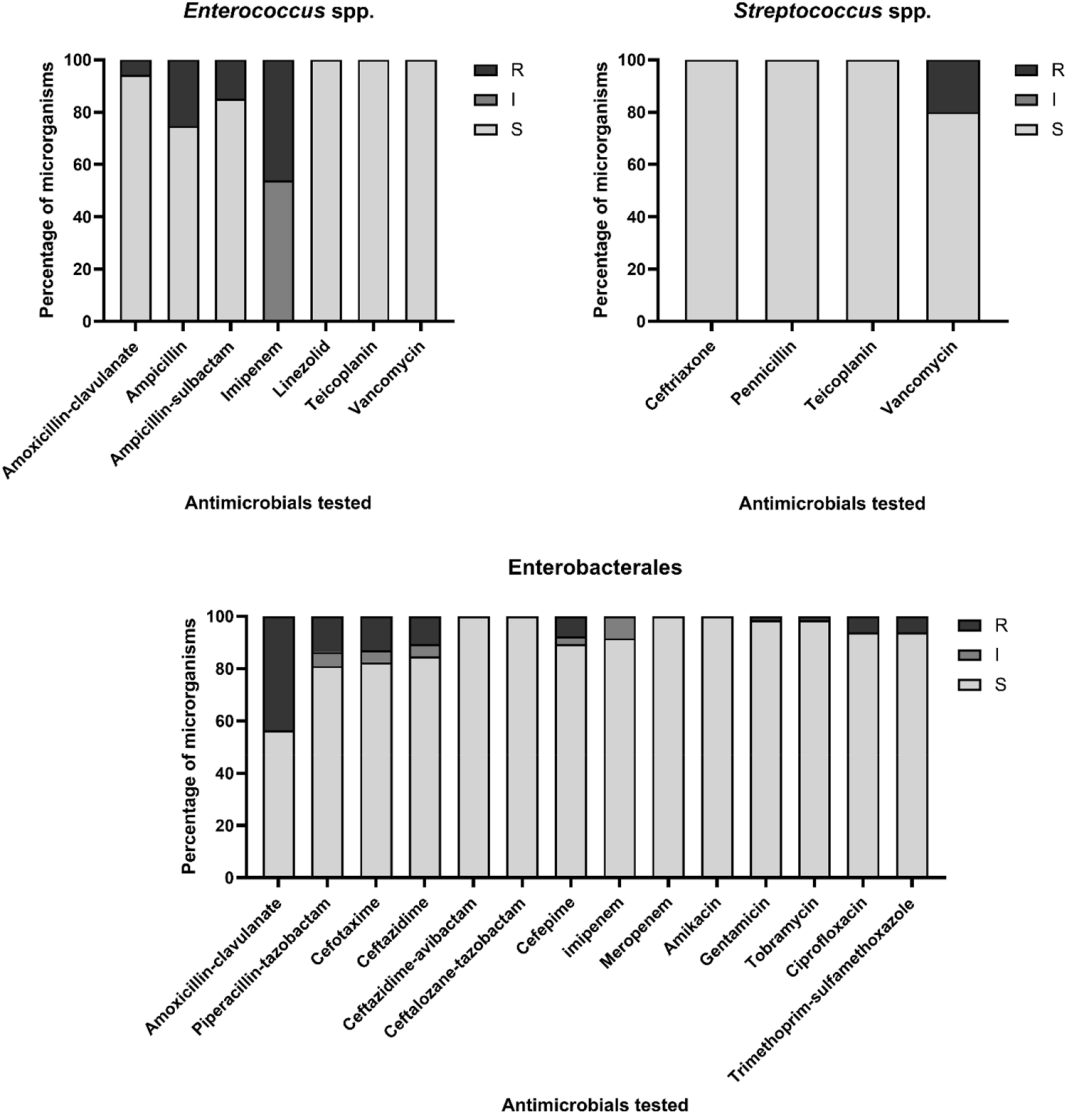
Susceptibility profile of *Enterococcus* spp., *Streptococcus* spp., or Enterobacterales stains identified in bile and stent samples. Resistant (R), susceptible standard dosing regimen (S), and susceptible, increased exposure (I) to antimicrobials tested.

Figure 2 shows the antimicrobial susceptibly profiles of the most represented isolates tested, grouped by *Enterococcus* spp, *Streptococcus* spp. and Enterobacterales. 56.0% of Enterobacterales and 5.8% of *Enterococcus* spp. were resistant to amoxicillin-clavulanate. Moreover, we found 13%, 10.7% and 7.6% of gram-negative bacteria, specifically *E. coli* and *K. pneumoniae,* resistant to cefotaxime, ceftazidime and cefepime, therefore showing a phenotype typical of ESBL (Extended spectrum beta-lactamase producer). The NG-Test CTX-M MULTI (NG Biotech) assay for ESBL detection showed that all the isolates were CTX-M-multi producers. Moreover, 13.7% of gram-negative bacteria were resistant to piperacillin-tazobactam. Interestingly, 15 of 22 patients in the study had received amoxicillin clavulanate as prophylaxis before the procedure, and 14 of 22 patients had received the same antibiotic in the previous 6 months (Tables 1 and 2). This suggests that preventive antibiotic therapy may have contributed to the development of drug resistance, as the highest resistance rates were found for this antibiotic, especially for Enterobacterales (Fig. 2).

### Microbiota profiling of bile and stent samples

**Figure 3:**
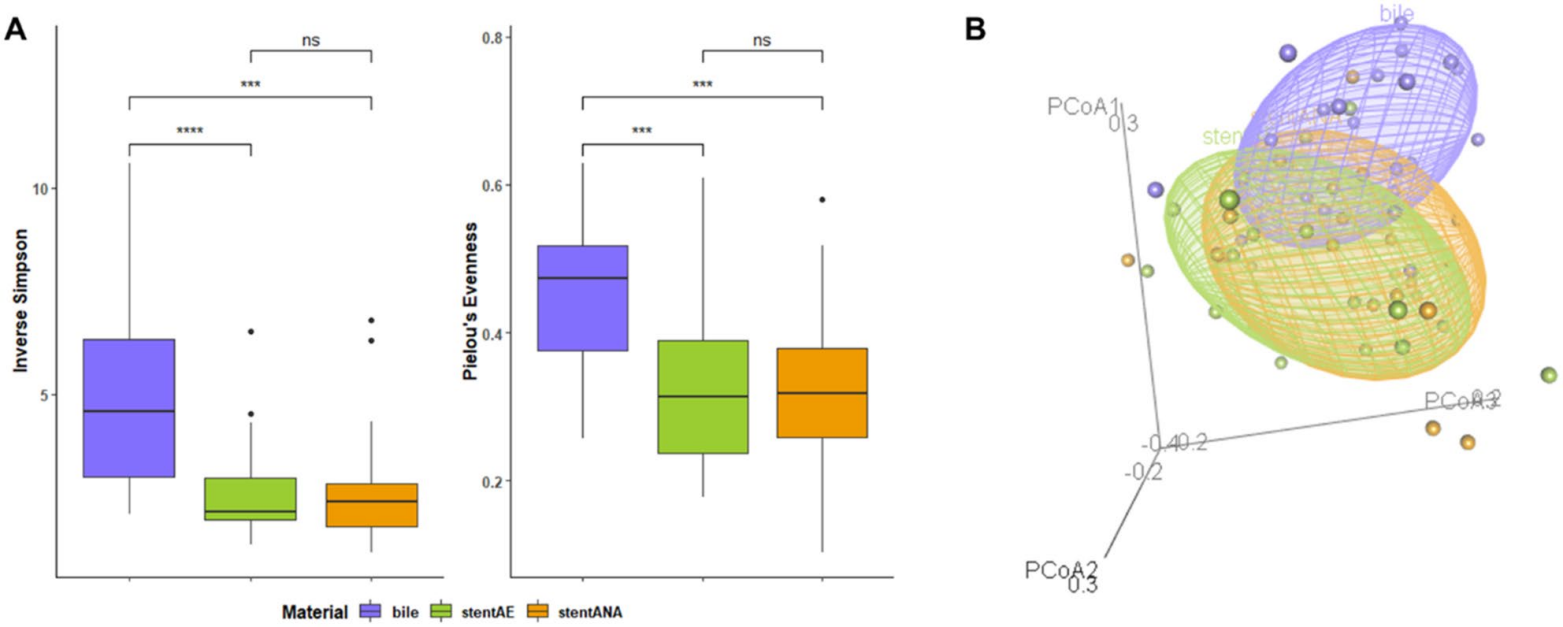
Alpha diversity and Beta diversity measurements of bile and stent specimens of our cohort. Alpha diversity metrics were reported as Inverse Simpson index and Pielou’s evenness (**A**). Statistical significance was assessed by using Kruskal–Wallis test. Beta diversity was measured by Weighted UniFrac distance and represented by Principal coordinate analysis (PCoA) (**B**). Permutational multivariate analysis of variance (PERMANOVA) was used to assess statistical significance.

Microbiome composition analysis of bile and stent samples exhibited a significant difference in α-diversity metrics (Inverse Simpson and Pielou’s Evenness index, Fig. 3A). Bile samples showed a higher diversity (average = 5.06, [median = 4.57, 2.11–10.58]; *p* < 0.0001) and major equitability (average = 0.45, [0.47, 0.25–0.62]; *p* = 0.0006) compared to microbial community recovered from stent samples cultured in aerobic or anaerobic conditions that accounted values of 2.64 [2.16, 1.37–6.52] and 0.33 [0.31, 0.17–0.60] or 2.70 [2.41, 1.18–6.79] and 0.32 [0.31, 0.10–0.58], respectively. No difference was detected between microbial compositions of the stent-derived cultures. Weighted Unifrac beta diversity matrix represented as principal coordinates analysis (PCoA) highlighted a different spatial distribution of bile samples compared to both stent derived samples (PERMANOVA analysis, *p* = 0.001) (Fig. 3B). Taken together these results suggested that a specific microbial population colonizes bile samples that may be partially hidden in stent derived cultures or due to overgrowth of some bacterial species independently by growth conditions.

Firmicutes and Proteobacteria represented the major phyla characterizing in both bile and stent derived samples (Supplementary Fig. 1). Firmicutes did not significantly differ among the three groups (*p* = *0.630*) and showed values of 27% [19, 0.5–75], 27% [16, 0.6–78] and 36% [29, 0.4–99] in bile, stentAE or stentANA, respectively. Proteobacteria were slightly more abundant in stent culture grown in aerobiosis and showed values of 68% [20–37] in comparison with bile samples (50% [50, 0.2–94]) or stent cultures grown in anaerobic condition (60% [67, 0.7–95]) (*p* = *0.089*). Conversely, Bacteroidetes showed values of 3.7% [1.0, 0.05–29], 2.1% [0.1, 0.02–13] and 1.0% [0.1, 0.01–11] in bile, stentAE or stentANA, respectively (*p* = *0.007*). Interestingly, Fusobacteria were significantly higher in bile samples (*p* < *0.001*) where reached a mean value of 12% [11, 0.1–34] and of 1.4% [0.2, 0.01–16] or 0.9% [0.3, 0.01–10] in stentAE or stentANA, respectively. Similarly, Actinobacteria were mostly detected in bile samples with a mean value of 2.4% that was 20 times higher than values detected in in stent samples (*p* < *0.001*).

**Figure 4:**
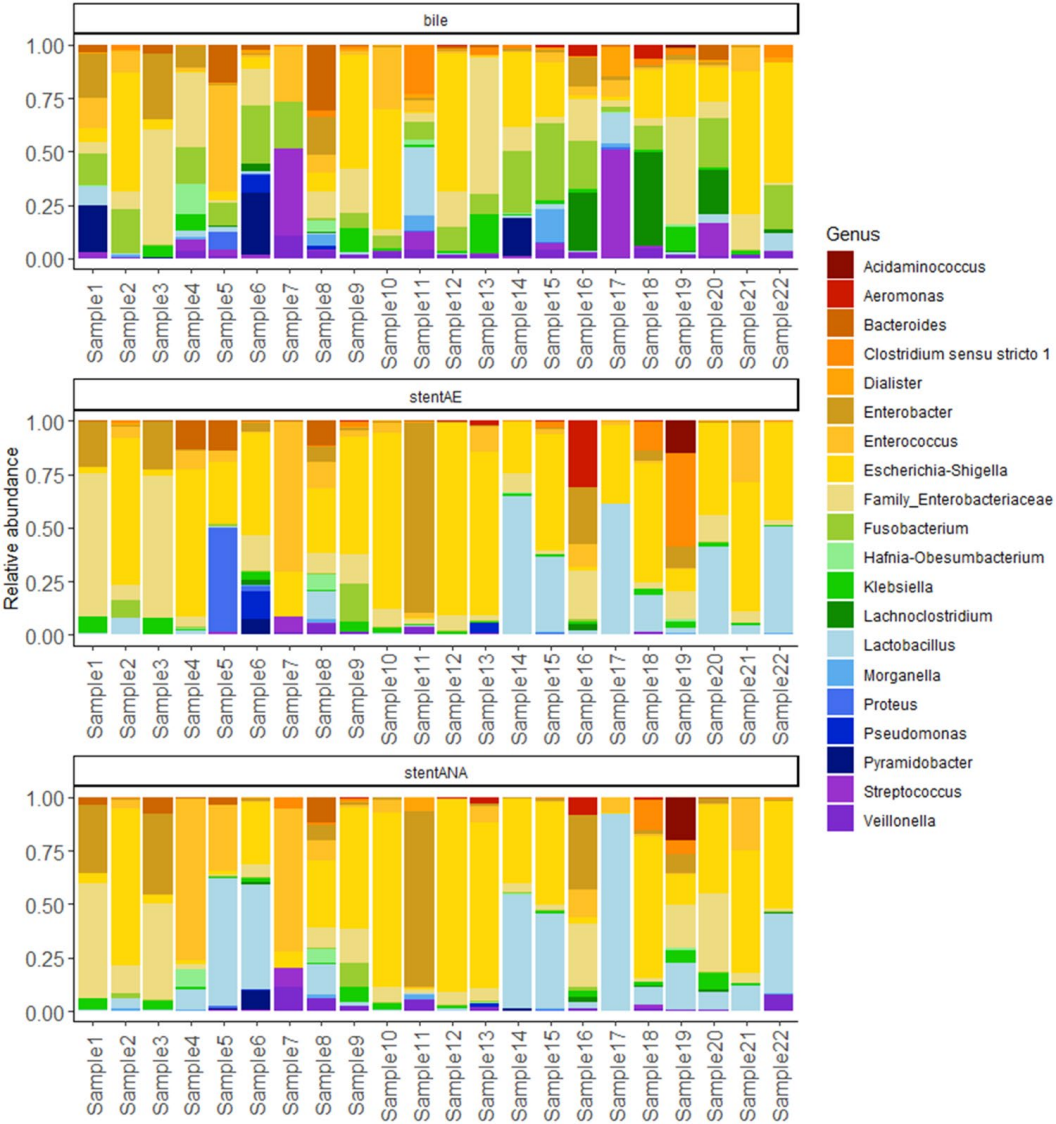
Relative abundances of the 20 most representative genera that compose the bile and stent bacterial community of our cohort. Each bar plot shows a single sample.

At genus level, *Escherichia-Shigella* and *Lactobacillus* genera were the most abundant of the top 20 genera (Fig. 4). Although not statistically significant, Escherichia-Shigella showed values of 21% [8.5, 0.1–65], 40% [43, 0.7–83] and 34% [35, 0.4–82] in bile, stentAE or stentANA, respectively. Conversely, *Lactobacillu*s genus was found more abundant in stent-derived cultures, 13% [1.6, 0.01–64] and 18% [8.4, 0.1–91], compared to bile samples (2.6%, [0.9, 0–20]). These results showed as Proteobacteria commonly colonize bile and may exert a key role as stent contamination and colonization. *Lactobacillus* (Firmicutes) genus retrieved from stent samples suggests a significant ability to colonize these medical devices, even though a major relative abundance may be dependent on culture condition enrichment.

**Figure 5:**
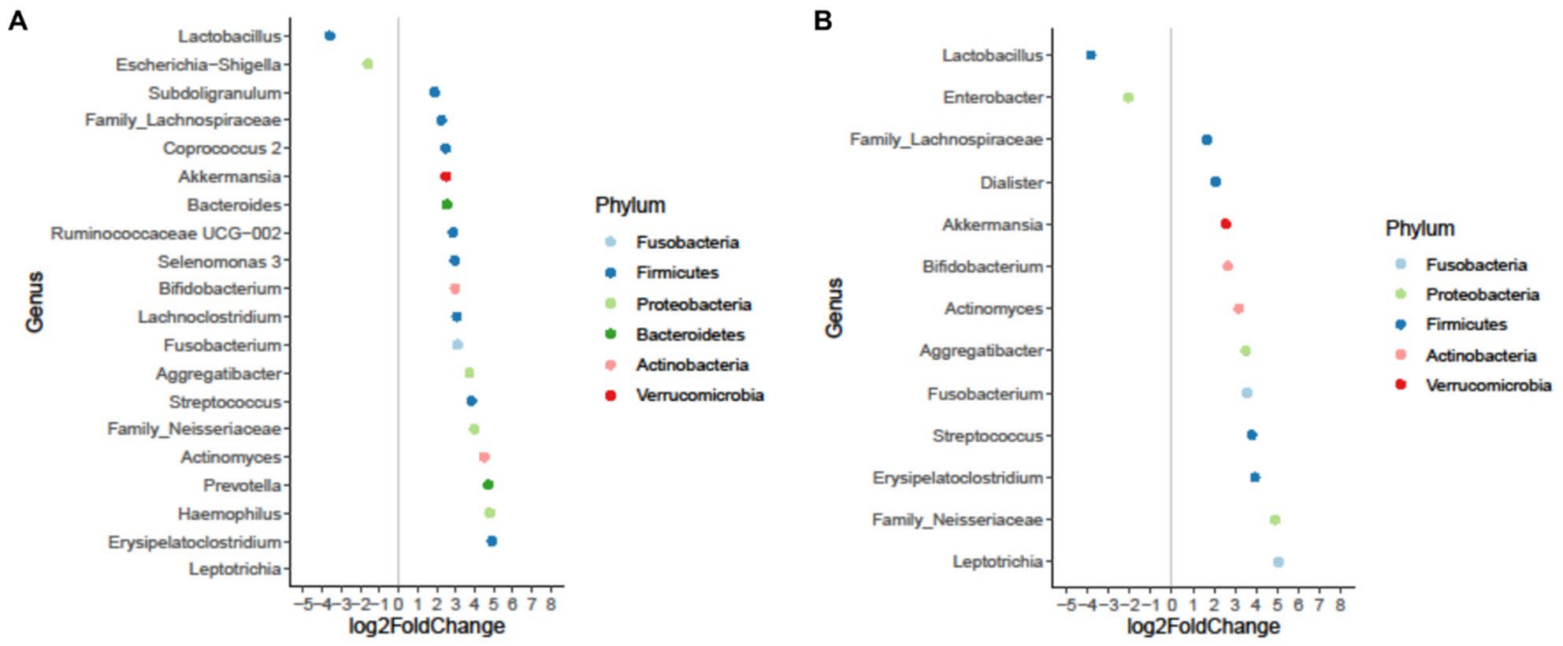
Differential abundances between bile samples and aerobic (panel **A**) /anaerobic (panel **B**) stents. The analysis of differentially abundant taxa was assessed using the DESeq2 package. In all analyses, a *P* value < 0.05 was set as the statistical significance threshold. Positive values of log2 Fold change represent genera significantly more abundant in aerobic/anaerobic stents.

DeSeq2 analysis was used to evaluate microbial biomarkers that were differentially abundant among bile and aerobic/anaerobic stents. The analysis revealed that *Lactobacillus* and *Enterobacter* genera were identified as biomarkers in stent samples than bile samples, suggesting a possible role in biofilm formation and stent occlusion (Fig. 5).

## Discussion

ERCP with biliary stenting has several therapeutic indications, including palliation for patients with advanced stage pancreatic or biliary tract cancer as well as BBSs^1^.

Self-expanding metal stents (SEMS) were designed to overcome the rapid clogging of plastic stents. The ideal solution in terms of cost and invasiveness would be a plastic stent that remains patent for 6 months, whether for benign biliary strictures (BBSs) or malignant biliary strictures. However, the goal of extending stent indwell time, reducing the number of required procedures, and avoiding cost increases has yet to be achieved.

The formation of biliary sludge is recognized as the main cause for stent cloggin^10,14^. Although the formation of biliary sludge may be a multifactorial event, bacterial biofilm creation and bacterial colonization are recognized as a leading factor in biliary stent blockage^10,24^.

**Figure 6:**
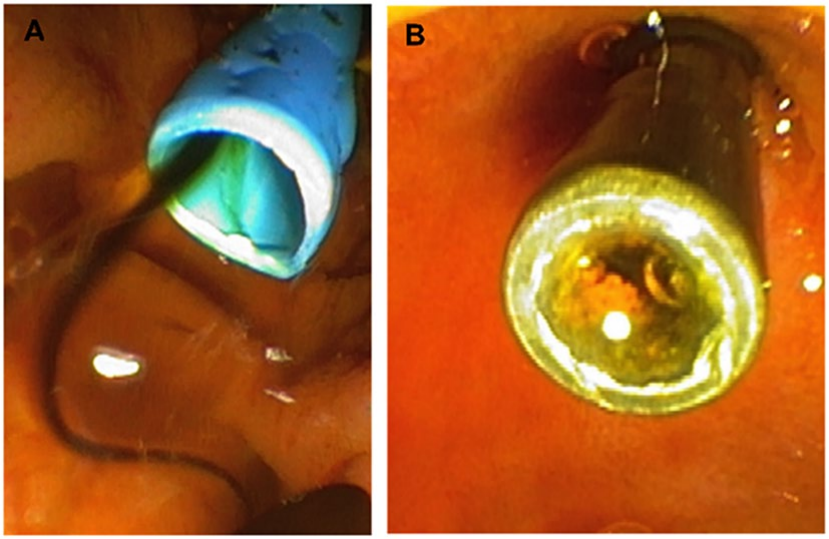
Representative images of a newly inserted plastic stent (**A**) and a removed stent (**B**) due to occlusion caused by sludge buildup and subsequent microbial biofilm development.

In more details, the first step in the clogging process is the microbial adhesion to the inner surface of stents, which leads to the creation of biofilms^10,25^ (Fig. 6). During ERCP and before stents placement a biliary sphincterotomy is done. This weakness the antimicrobial barrier related to the sphincter of Oddi thus allowing microorganisms ascending migration and colonization^24,25^. Some authors suggest placing stents without performing sphincterotomy and above the sphincter of Oddi, to reduce stents malfunction, but however also in these series stent obstruction was reported^26,27^. A biofilm is an organized community of microbes encased in a self-made exopolysaccharide matrix including proteins and other polymers that grows on a solid synthetic surface^14,24,25^. Most of biofilms are polymicrobial communities, which act synergistically in the biofilm growth, which gradually becomes thicker and drives stent blockage^14,25,28,29^.

As stent obstruction is a major issue in the treatment of biliary strictures, some stent modifications (i.e. design changes, especial coatings and new biomaterials) have been proposed to reduce the biofilm development and extend patency duration, but no definitive data are available to support their introduction in clinical practice^2,14^.

As the biofilm formation is a driving force in the stent blockage, a better knowledge of the leading microorganisms involved is crucial in the development of strategies to prevent this adverse event.

There are several studies that tried to detect the microbial species composition in biliary stents by traditional cultural methods^12,24,30–34^, while few others by molecular techniques^25,35,36^. All of them analysed the composition of the stents but not from bile, assuming that the composition of the two samples were similar^12^.

In this study, we analysed bile and corresponding stent samples to determinate the microbial population that is mainly involved in the stent occlusion process. In accordance with the previous reported data, the most common aerobic bacteria isolated from bile and stents samples and identified by culturing techniques followed by MALDI-TOF were the gram-positive *Enterococcus* species, especially *E. faecalis* and *E. faecium* and the gram-negative bacteria *E. coli* and *K. pneumoniae*^10,24,34^*.* Among yeast, *Candida* species represented the most isolated yeast, and *Candida albicans* resulted to be the predominant species, as already reported^24,34^*.* The anaerobic bacteria identified vary among the different studies. A study from Leung et al.^32^ reported the early attachment of *Clostridium perfrigens*, *Clostridium bifermentas* and *Bacteroides fragilis* to the stents as fundamental step toward the stent occlusion process; another study reported the presence of *Bacteroides* spp., *Prevotella* spp. and *Veillonella* spp. as predominant anaerobic species^24^ while another study reported *Fusobacterium* spp. and *Veilonella* spp*.* as the most observed anaerobic species analysed by SSCP (single strand conformation polymorphism)^35^.

Cultural techniques revealed four distinct anaerobic bacteria species: *Clostridium* spp., *Bacteroides* spp., *Bifidobacterium* spp., and *Veilonella* spp (Table 2). These were the most encountered species, but *Fusobacterium* spp. was also observed.

This study used a metagenomic approach to comprehensively assess the microbial composition and functional potential of bile and stent samples from patients with biliary benign strictures. The data confirmed the results obtained by cultural techniques, with Firmicutes (to which belong *Enterococcus* spp. and *Clostridium* spp.) and Proteobacteria (to which belong *E. coli* and *K. pneumoniae*) that represent the most observed phyla in either bile or stent samples.

DeSeq analysis revealed that *Lactobacillus* spp. and Enterobacterales were more abundant in stent samples then in bile samples, confirming the trend observed by cultural approach, in which the percentage of *Lactobacillus* spp*.* isolated from stents is slightly higher than the percentage of isolates in bile (11.7% and 8.5%, respectively).

Scheithauer and collaborators reported a high prevalence of *Lactobacillus* spp. in biliary stent samples, speculating that as *Lactobacillus* spp are present either in the ileum and duodenum^37^, it is probable that they are major colonizer of the stents and involved in the process of stent occlusion.

To our knowledge, this is the first pilot study that explores the microbial composition of both bile and corresponding stents samples by two different approaches: traditional cultural techniques and metagenomic analysis. These results contribute to a better comprehension of the main microorganisms involved in biliary stent occlusion allowing the study of new anti-adhesive stent composition to inhibit microbial biofilm and stent blockage.

## Conclusion

**Figure 7:**
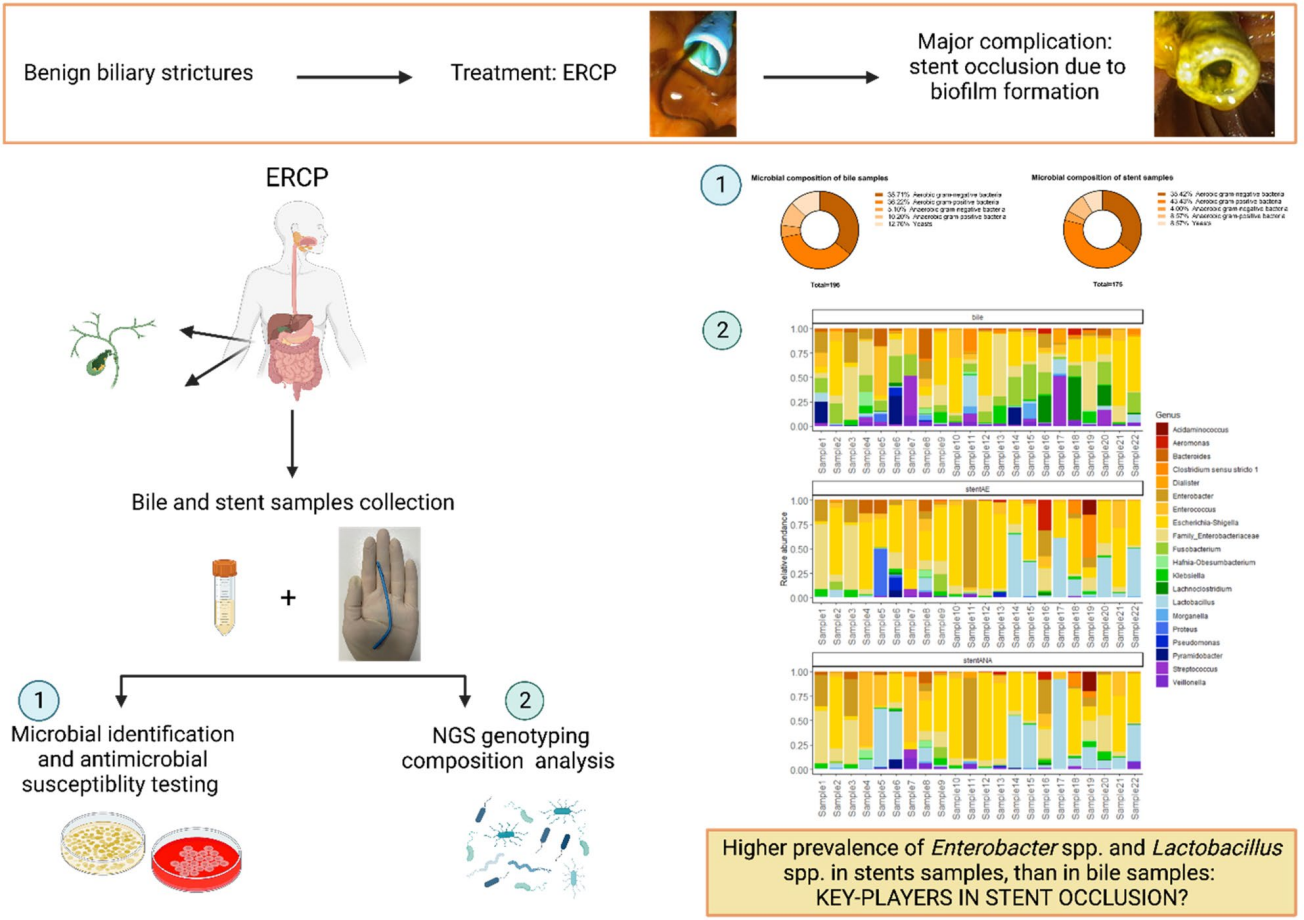
Graphical representation of the aims and results of the study.

This pioneering metagenomic study sheds light on the microbial composition and functional potential within biliary benign strictures, advancing our understanding of the complex interplay between microbial communities and the biliary tract microenvironment. The findings underscore the necessity of a multi-sample approach encompassing both bile and stent samples in future studies aiming to elucidate the role of microbes in biliary strictures. Ultimately, this research has the potential to inform the development of innovative diagnostic tools and targeted therapeutic strategies for patients affected by biliary benign strictures. To succinctly summarize and elucidate the study's objectives see Fig. 7.

### Supplementary Information

Supplementary Information.

## Data Availability

The datasets generated and/or analyzed during the current study are not publicly available but are available from the corresponding author on reasonable request.
