# Supplementary material for: Pilot study on cultural and metagenomic analysis of bile and biliary stentslead to unveiling the key players in stent occlusion
Source: Sci Rep. 2024 Feb 9;14:3344. doi: 10.1038/s41598-024-51480-2 (PMC10858256; doi:10.1038/s41598-024-51480-2)

Supplementary material

Pilot study on cultural and metagenomic analysis of bile and biliary stents lead to unveiling the key players in stent occlusion

Margherita Cacaci^1,2^*, Flavio De Maio^2^*, Maria Valeria Matteo^3,4^, Brunella Posteraro^1,5^, Maura Di Vito^1^, Giulia Menchinelli^2^, Andrea Tringali^3,4^, Francesca Romana Monzo,^2^ Riccardo Torelli^2^, Guido Costamagna^3,4^, Cristiano Spada^3,4^, Francesca Bugli^1,2^, Maurizio Sanguinetti ^1,2#^, Ivo Boskoski^3,4#^.

^1^Department of Basic Biotechnological Sciences, Intensive and Perioperative Clinics, Università Cattolica del Sacro Cuore, 00168 Rome, Italy^.^

^2^Department of Laboratory and Infectious Sciences, Fondazione Policlinico Universitario A. Gemelli IRCCS, Largo A. Gemelli 8, 00168 Rome, Italy

^3^Digestive Endoscopy Unit, Fondazione Policlinico Universitario Agostino Gemelli IRCCS, Roma, Italy.

^4^Center for Endoscopic Research Therapeutics and training (CERTT), Università Cattolica del Sacro Cuore, Roma, Italy.

^5^Department of Abdominal and Endocrine Metabolic Medical and Surgical Sciences, Fondazione Policlinico Universitario A. Gemelli IRCCS, 00168 Rome, Italy.

Corresponding authors: Francesca Bugli, Francesca.Bugli@unicatt.it

*Equally contributed

^#^ Equally contributed

Table S1. Detailed baseline characteristics.

| **Patient** | **Age (years)** | **Sex** | **Etiology of BBSs** | **Number of previous stent placement/exchange** | **Clinical setting of stents extractions** | **Indwelling time of stents (days)** | **Antibiotic prophylaxis** | **Antibiotic therapy in the previous 6 months** |
| --- | --- | --- | --- | --- | --- | --- | --- | --- |
| 1 | 60 | M | Biliary stricture after cholecystectomy | 2 | elective | 83 | Amoxicillin/Clavulanic Acid | Amoxicillin/Clavulanic Acid # |
| 2 | 80 | M | Biliary stricture after cholecystectomy | 4 | elective | 95 | Amoxicillin/Clavulanic Acid | Amoxicillin/Clavulanic Acid # |
| 3 | 73 | M | Biliary stricture after cholecystectomy | 3 | elective | 107 | Amoxicillin/Clavulanic Acid | Amoxicillin/Clavulanic Acid # |
| 4 | 51 | M | Biliary stricture after cholecystectomy | 3 | elective | 134 | N/A | N/A |
| 5 | 63 | M | Biliary stricture after severe acute pancreatitis | 1 | elective | 660 | N/A | N/A |
| 6 | 82 | M | Idiopathic biliary stricture | 6 | elective | 210 | Amoxicillin/Clavulanic Acid | Amoxicillin/Clavulanic Acid # (7 months before) |
| 7 | 74 | F | Idiopathic biliary stricture | 4 | elective | 120 | N/A | N/A |
| 8 | 71 | F | Biliary stricture after cholecystectomy | 3 | elective | 120 | Amoxicillin/Clavulanic Acid | Amoxicillin/Clavulanic Acid # |
| 9 | 49 | M | Biliary stricture after cholecystectomy | 2 | elective | 84 | Amoxicillin/Clavulanic Acid | Amoxicillin/Clavulanic Acid # |
| 10 | 51 | M | Biliary stricture after cholecystectomy | 4 | elective | 195 | Piperacillin/Tazobactam** | Not available |
| 11 | 51 | F | Biliary stricture after cholecystectomy | 3 | elective | 107 | Amoxicillin/Clavulanic Acid | Amoxicillin/Clavulanic Acid # |
| 12 | 42 | F | Biliary stricture after cholecystectomy | 2 | elective | 103 | None | None |
| 13 | 61 | F | Biliary stricture after cholecystectomy | 1 | elective | 148 | Amoxicillin/Clavulanic Acid | None |
| 14 | 65 | F | Biliary stricture after cholecystectomy | 3 | elective | 90 | Amoxicillin/Clavulanic Acid | Amoxicillin/Clavulanic Acid # |
| 15 | 78 | M | Biliary stricture after cholecystectomy | 2 | elective | 98 | Amoxicillin/Clavulanic Acid | Amoxicillin/Clavulanic Acid ‡ |
| 16 | 67 | F | Biliary stricture after cholecystectomy | 4 | elective | 138 | Amoxicillin/Clavulanic Acid | Amoxicillin/Clavulanic Acid # |
| 17 | 52 | M | Biliary stricture after cholecystectomy | 5 | emergency (cholangitis) | 264 | Ciprofloxacin +  Metronidazole* | Ciprofloxacin +  Metronidazole ‡ |
| 18 | 68 | M | Biliary stricture after cholecystectomy | 2 | elective | 104 | Amoxicillin/Clavulanic Acid | Piperacillin/Tazobactam ‡ |
| 19 | 64 | F | Biliary stricture after cholecystectomy | 2 | elective | 164 | Amoxicillin/Clavulanic Acid | Amoxicillin/Clavulanic Acid # |
| 20 | 68 | M | Idiopathic biliary stricture | 1 | emergency (cholangitis) | 60 | Ceftriaxone* | Piperacillin/Tazobactam ‡ |
| 21 | 70 | F | Biliary stricture after cholecystectomy | 22 | emergency (obstructive jaundice) | 191 | Amoxicillin/Clavulanic Acid | Amoxicillin/Clavulanic Acid ‡ |
| 22 | 65 | M | Biliary stricture after cholecystectomy | 3 | elective | 150 | Amoxicillin/Clavulanic Acid | Amoxicillin/Clavulanic Acid # |
| * Administered twice daily during hospitalization  ** Administered 3 times a day during hospitalization  # Prophylaxis in the previous ERCP  ‡ Administered for 7-10 days concomitantly with the previous ERCP  N/A: not available. | | | | | | | | |

Figure S1: Relative abundances of the 8 most representative phyla that compose the bile and stent bacterial community of our cohort. Each bar plot shows a single sample.


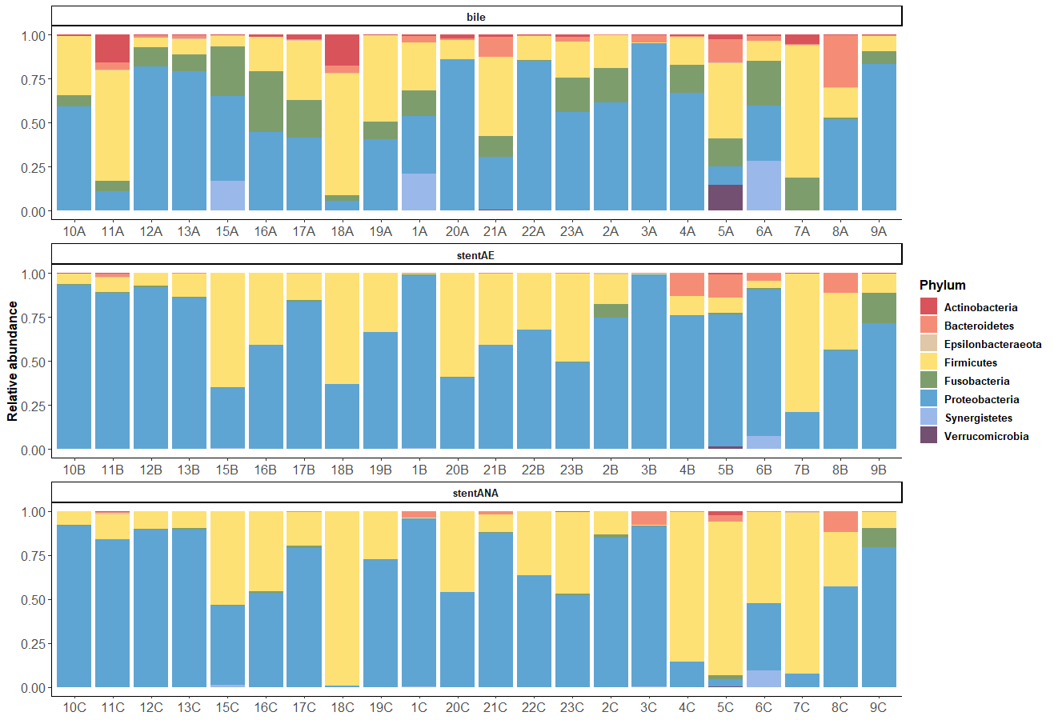

Supplement: Supplementary file 1 — Supplementary Information. [file 41598_2024_51480_MOESM1_ESM.docx]
